# miR-29b-3p promotes progression of MDA-MB-231 triple-negative breast cancer cells through downregulating TRAF3

**DOI:** 10.1186/s40659-019-0245-4

**Published:** 2019-07-26

**Authors:** Bao Zhang, Dattatrya Shetti, Conghui Fan, Kun Wei

**Affiliations:** School of Biology and Biological Engineering, South China University of Technology, Guangzhou, 510640 People’s Republic of China

**Keywords:** miR-29b-3p, Triple negative breast cancer, Cytoskeleton, TRAF3, NF-κB

## Abstract

**Background:**

Breast cancer is the second common malignant cancer among females worldwide. Accumulating studies have indicated that deregulation of miRNA expression in breast cancer will contribute to tumorigenesis and form different cancer subtypes. However, the reported studies on miR-29b-3p-regulated breast cancer are limited so far. Herein, we investigated the role and mechanism of miR-29b-3p in the triple negative breast cancer cell line MDA-MB-231.

**Methods:**

The relative miR-29b-3p expression in different breast cancer cell lines were determined by qRT-PCR. CCK8 and colony formation assay were used to determine the influence of miR-29b-3p on cell proliferation. Migration assay and invasion assay were performed for cell migration and invasion respectively. To study the cell integrity immunofluorescence was performed. TUNEL assay, flow cytometry assay, hoechst staining and western blot were conducted to determine the influence of miR-29b-3p inhibitor on cell apoptosis. TRAF3 was found to be the target gene of miR-29b-3p using bioinformatics predictions. Dual-luciferase assay was performed to determine the relative luciferase activity in NC, miR-29b-3p mimic, miR-29b-3p inhibitor with TRAF3 3′-UTR wt or TRAF3 3′-UTR mt reporter plasmids. The proteins expression of NF-κB signaling pathway in MDA-MB-231 after transfection with NC, miR-29b-3p mimic, miR-29b-3p inhibitor were determined by western blot.

**Results:**

The miR-29b-3p expression was significantly increased in MDA-MB-231 compare with MCF-10A. miR-29b-3p inhibitor reduced the cell viability of MDA-MB-231 and inhibited cell migration and invasion. Cell cytoskeleton integrity destroyed after miR-29b-3p inhibitor treatment. Furthermore, we identified the mechanism and found miR-29b-3p targets the TRAF3 and activates NF-κB signaling pathway.

**Conclusions:**

From the above studies, our results indicated that miR-29b-3p acts as a promoter for the development of MDA-MB-231.

## Background

Breast cancer is considered to be one of the most common malignant cancer among females worldwide. According to the American Cancer Society, breast cancer accounts for 30% of all new cancer cases and is a major cause of cancer-related mortality in females [1–3]. In China, around 12.2% breast cancer cases have been diagnosed and about 9.6% death cases have been reported from breast cancer worldwide [4]. In addition, the 5-year survival rate (68%) [5, 6] is much lower than that in the United States (90%) [2]. Attributed to improvements in early diagnosis and follow-up on effective treatments the mortality rate of breast cancer has decreased since 1987. However, the incidence of breast cancer has been increasing year by year in developing countries [2]. In China, incidence rate is growing at more than twice the rate of global growth, especially in urban areas [4]. Triple negative breast cancer (TNBC) is a subtype of breast cancer which lack of estrogen receptor, progesterone receptor and low expression of human epidermal growth factor receptor 2 (HER 2) [7–9]. Due to these, the prognosis was worse than other subtypes of breast cancer. Therefore, finding new biomarkers and developing effective therapeutic strategies for TNBC is instantly needed.

microRNA is a kind of small, 19–22 nucleotides in length, non-coding RNA which play a crucial role in the regulation of gene expression by binding to the 3′-untranslated region (UTR) of target mRNA [10–12]. Recent studies have demonstrated that miRNAs involved in many biological processes including cell proliferation, differentiation, apoptosis, development of organisms and pathogenesis of cancer [13–16]. It has been reported that miR-15a/miR-16-1 cluster act as a tumor suppressor in chronic lymphocytic leukemia (CCL) because of this they are often been deleted on the chromosome [17]. Zhang et al. [18] showed that miR-21 binds to PTEN and promotes tumor proliferation and invasion in gastric cancer. In breast cancer, many miRNAs levels are dysregulated, such as miR-210, miR-19b, miR-93 are downregulated and miR-10b, miR-145, miR-195 are upregulated, causing many functional disorders [19–22]. Therefore, miRNAs could be novel therapeutic molecular targets for breast cancer treatment.

miR-29b, one of miR-29 family members, has been reported to serve as a promoter among various kinds of disease. Zhang et al. [23] investigated that miR-29b is an effective positive regulator of adipogenesis which causes obesity. Maegdefessel et al. [24] reported that inhibition of miR-29b reduces murine abdominal aortic aneurysm development. Yang et al. [25] reported that miR-29b is a cell–cell adhesion regulator which promoted oral squamous cell carcinoma cell migration and downregulated CX3CL1. Liu et al. [26] found that miR-29b levels were significantly elevated in AML leukemia stem cells compared with non-leukemia stem cells and the miR-29b silencing resulted in increased Sp1 in AML cells, it is a promoter in acute myeloid leukemia. Langsch et al. [27] found that miR-29b protects NSCLC cells from extrinsic apoptosis and mediates NF-κB signaling in KRAS-Induced Non-Small Cell Lung Cancers. Jarline et al. identified the miRNAs related to variations in DNA repair capacity levels in BC cases. miR-29b-3p has a higher expression in BC cases with low DNA repair capacity levels which positively associated with BC risk [28]. The members of miR-29b family are miR-29b-3p, miR-29b-1-5p, miR-29b-2-5p, and they have the high similarity of mature sequences. Despite it, distinct functions were identified because of the different isoforms [29–32].

In this study, we investigated the role of miR-29b-3p expression in triple negative breast cancer cell. Furthermore, potential targets of miR-29b-3p was identified by bioinformatics analysis and dual-luciferase reporter assay. Our study revealed that inhibition of miR-29b-3p drastically reduces cell proliferation, migration, invasion in TNBC. Moreover, inhibiting miR-29b-3p also induces apoptosis in TNBC through PARP cleavage. However, mechanism involved in the inhibition of miR-29b-3p and pathogenesis and metastasis yet to be studied.

## Materials and methods

### Cell culture and transfection

Human breast cancer cell lines MDA-MB-231, MCF-7, MDA-MB-453 and human epithelial cell line MCF-10A were purchased from Procell (Wuhan, China). MDA-MB-231, MCF-7, MDA-MB-453 were cultured in complete Dulbecco’s modified Eagle’s medium (DMEM, Gibco, USA) which supplemented with 10% fetal bovine serum (FBS, Gibco, USA), 1% penicillin streptomycin (100 U/mL penicillin and 100 mg/mL streptomycin, P&S, Gibco, USA). MCF-10A was cultured in human epithelial cell complete medium (Procell, Wuhan, China) which containing DMEM/F12, horse serum, growth additive and P&S. All cell lines were cultured at 37 °C in 5% CO_2_ atmosphere. miR-29b-3p mimics and miR-29b-3p inhibitor, negative control, negative control inhibitor were purchased from GenePharma (Suzhou, China). To conduct cell transfection, cells were firstly cultured to about 50–60% confluence. Then, Lipofectamine 2000 (Thermo Fisher Scientific, USA) was used and transfected according to the manufacturer’s manual.

### Total RNA extraction and qRT-PCR (quantitative real-time polymerase chain reaction)

Total RNAs were isolated from cells after 24 h transfection according to the manufacturer’s protocol (AM1560, Invitrogen, USA), cDNA synthesis and miRNA expression were quantified using Mir-X™ miRNA qRT-PCR SYBR^®^ Kit (Takara, Japan) and Applied Biosystems 7500 (Thermo Fisher Scientific, USA) real time device under the standard thermal condition: 95 °C for 30 s, 40 cycles of 95 °C for 10 s and 60 °C for 30 s, followed by 95 °C for 10 s, 65 °C for 60 s and 97 °C for 1 s. All results were normalized to U6. Using the − 2^△△Ct^ method to identify the miRNA relative expression levels. Primer sequences: U6, forward primer: 5′-CTCGCTTCGGCAGCACA-3′; Reverse primer: 5′-AACGCTTCACGAATTTGCGT-3′; miR-29b-3p, forward primer: 5′-TAGCACCATTTGAAATCAGTGTT-3′, Reverse primer: mRQ 3′ Primer (CAS: 638313, Takara, Japan). Three independent experiments were performed for each reaction in triplicate.

### Doxorubicin treatment

Doxorubicin hydrochloride was purchased from Macklin (CAS: 25316-40-9, Shanghai, China). For Doxorubicin treatment, we prepared the Doxorubicin stocking solution at 1 mM concentration in ultrapure water. Then, different concentration of Doxorubicin (10 μΜ, 1 μΜ, 0.1 μΜ, 0.01 μΜ, 0.001 μΜ) were dissolved in medium and treated for 24 h, 48 h and 72 h and CCK8 assay was performed, and IC50 of DOX was used for treatment to identify the miRNA relative expression.

### Cell proliferation assay

Cell proliferation of MDA-MB-231 was detected using Cell Counting Kit-8 (CCK8) (Beyotime, Shanghai, China). Briefly, 8000 cells per well were seeded in 96-well plate with 100 μL complete medium and cultured for 24 h. After Doxorubicin or transfection treatment, 100 μL medium containing 10 μL CCK8 was added to the wells and incubated for 4 h later absorbance was measured using EnSpire Multimode Plate Reader (PerkinElmer, Waltham, Massachusetts, USA) at 450 nm. Each group in triplicate.

### Colony formation assay

Single cell suspension was obtained by trypsinization after transfection for 24 h. 500 cells were seeded in 6-well plate and cultured it for 2 weeks with complete medium in 5% CO_2_ incubator. After colony formation, 4% paraformaldehyde (PFA) (Biosharp, Hefei, China) was used to fix the cells for 20 min and stained with 1% crystal violate (KeyGen BioTECH, Jiangsu, China) for 15 min at room temperature. The number of colony- forming units were calculated in three random wells.

### Migration assay

To study the migration ability of MDA-MB-231 migration assay were conducted. Briefly, 5 × 10^5^ MDA-MB-231 cells were seeded in 24-well and culture overnight. After 80–90% confluence transient transfection was performed for 24 h, later 1 μL sterile tip was used to make a straight wound. Scratch photo was taken at 0 h, 6 h and 12 h by microscope (Olympus, Japan). Distance migration was measured by calculating the area of the wound using Image J software and represented in the form of bar graph.

### Invasion assay

To detect the invasive ability of MDA-MB-231 invasion assay was performed. After 24 h of transient transfection, 2.5 × 10^4^ cells were harvested and seeded in the 8 μm pore size upper Transwell chamber (Corning, USA) coated with Matrigel (CAS:356234, Corning, USA), which cultured in basic medium. The optimal coating concentration ratio was DMEM serum-free medium: Matrigel = 8:1 (V/V), and 2 h incubation at 37 °C. 900 μL complete medium containing 5% FBS was added into the lower chamber as a chemoattractant. After 24 h incubation cells were fixed with 4% paraformaldehyde and stained with 1% crystal violate. Migratory cells in lower chamber were counted from each well and photographed at 20× (Olympus, Japan). The experiment was repeated three times.

### Immunofluorescence

To study the cytoskeleton reorganization in breast cancer cell line MDA-MB 231 immunofluorescence technique was performed. 1 × 10^5^ MDA-MB-231 cells were seeded on cover slips in 24 well plate. After 36 h of NC, miR-29b-3p mimics and miR-29b-3p inhibitor treated cells were fixed with 4% paraformaldehyde for 15 min at room temperature and then washed with PBS twice. Later, the cells were washed with a quenching solution (0.1% glycine in PBS) twice, permeated with 0.1% Triton X-100 for 10 min and blocked using a blocking solution (10% FBS in PBS) for 1 h at room temperature. Later the cells were incubated for overnight with primary antibody against α-Tubulin (anti α-Tubulin (11H10), 1:100, 2125, Cell Signaling Technology, USA) followed by secondary antibody Alexa Fluor 594 Conjugated goat anti-rabbit IgG (1:50, ZF-0516, ZSGB-Bio, Beijing, China). Nuclei were stained with 4,6-diamidino-2 phenylindole DAPI and the cover slip was mounted using anti-fluorescent quencher (Beyotime, Shanghai, China). Immunofluorescence image were captured under LSM 710 Laser scanning confocal microscope (Carl Zeiss, German). Digital images were optimized for image resolution (final resolution 300 dpi), brightness, and contrast using Adobe Photoshop 7.0 (Adobe Systems, San Jose, CA, USA). No alteration was made in image such as adding or removal of image details.

### Hoechst staining

To study the nuclear morphology of the MDA-MB-231 and MCF-7, Hoechst staining was performed. 1 × 10^5^ MBA-MB-231 and MCF-7 cells were seeded on the cover slip in 24 well plate and transfected with NC, miR-29b-3p mimics and miR-29b-3p inhibitor for 36 h. The cells were fixed for 20 min with 4% PFA then washed with PBS, incubated with 0.3% Triton-X 100 for 5 min. Later cells were washed and 100 μL Hoechst 33258 (Beyotime, Shanghai, China) was added to cells and incubated for 30 min in dark. The cover slip was mounted using anti-fluorescent quencher (Beyotime, Shanghai, China). Photographed under the LSM 710 Laser scanning confocal microscope at 40× (Carl Zeiss, German).

### TUNEL assay

1 × 10^5^ MDA-MB-231 cells were seeded in 24-well plate and transfected with NC, miR-29b-3p mimics and miR-29b-3p inhibitor for 36 h. The cells were fixed with 4% paraformaldehyde for 30 min then wash with PBS. Later 0.3% Triton-X 100 in PBS was added and incubated for 5 min. One-step TUNEL apoptosis detection kit (Beyotime, Shanghai, China) was used to estimate the apoptosis efficiency according the manufacture’s instruction. In brief, cells were incubated with 50 μL TUNEL reaction buffer in a 37 °C humidified chamber for 1 h, nucleus was counterstained with DAPI for 1 min at room temperature in dark later mounted with anti-fade mounting medium. Stained apoptotic cells were visualized at 20× by LSM 710 Laser scanning confocal microscope (Carl Zeiss, German).

### Flow cytometry assay

Annexin V/propidium iodide staining for apoptosis was conducted using Annexin V-FITC Detection kit (DojinDo, Japan) according to the manufacturer’s instructions. Briefly, 1 × 10^6^ MDA-MB-231 cells were seeded in 6 well plate after 24 h the cells were transfected with NC, miR-29b-3p mimics and miR-29b-3p inhibitor. The cells were trypsinized and single cells suspension was added with 5 μL Annexin V-FITC followed by 5 μL PI solution in dark and incubated for 15 min. Soon 400 μL Annexin V Binding Solution was added and analyzed by BD Accuri C6 Plus (BD) flow cytometer. Necrotic, early and late apoptosis cells were identified by BD Accuri C6 Plus software.

### Dual-luciferase reporter assay

MDA-MB-231 cells were seeded into 24-well plates and after 24 h incubation the confluence reaches to 60–70%. Psi-CHECK2/TRAF3-3′-UTR wt and Psi-CHECK2/TRAF3-3′-UTR mutant reporter plasmids were constructed in advanced. According to the manufacturer’s instruction, MDA-MB-231 cells were transiently co-transfected with miR-29b-3p mimics or miR-29b-3p inhibitor together with 0.1 μg psi-CHECK2/TRAF3-3′-UTR wt or psi-CHECK2/TRAF3-3′-UTR mutant reporter plasmids using Lipofectamine 2000. After 48 h, Dual-luciferase Reporter Assay System (CAS: E1910, Promega, USA) was used to detect firefly and Renilla luciferase activities and recorded using GloMax 96 Microplate Luminometer (Promega, USA).

### Western blot

Breast cancer cell line MDA-MB-231 (1 × 10^6^ cells/each well in 6-well plates) was transfected with NC, miR-29b-3p mimics and miR-29b-3p inhibitor for 48 h. Cell lysates were collected using 1 mL RIPA (Solarbio, Beijing, China) with 10 μL PMSF. Protein concentration of cell lysates were measured with a BCA Protein Assay Kit (Sangon Biotech, Shanghai, China). Western blot was carried out by separating 40 µg of protein by sodium dodecyl sulfate polyacrylamide gel electrophoresis (SDS-PAGE) and electro-transferred onto a polyvinylidene difluoride (PVDF) membrane (240 mA, 4 h). The membranes were blocked with 5% non-fat milk and washed with TBST buffer and then incubated with primary antibody Cleaved PARP (Asp214) (D64E10), NF-κB p65 (D14E12), phospho-NF-κB p65 (ser536), IκB-α (44D4), phospho-IκB-α (14D4), (Cell Signaling Technology, USA) at a dilution of 1:1000 in Primary Antibody Dilution Buffer (Beyotime. Shanghai, China) at 4 °C overnight. Next, membrane treated with primary antibody were washed 3 times with TBST and incubated with an anti-rabbit HRP-linked antibody at a 1:5000 dilutions for 1 h at room temperature, β-Actin was used as an internal reference. The signals were visualized by Immobilon Western Chemiluminescent HRP Substrate (Millipore Corporation, Billerica, MA, USA) with the Amersham Imager 600 imagers (GE healthcare Life science, Pittsburgh, USA).

### Statistical analysis

Each experiment was repeated at least three times. All data were analyzed by using GraphPad Prism 6.01 and presented as mean ± S.E.M. Differences between groups were considered significant at p < 0.05. We evaluated it by using the independent t-test for the comparison of two samples and using a one-way ANOVA test for the comparison of more the two samples.

## Results

### miR-29b-3p was dysregulated in MDA-MB-231 and MCF-7

**Fig. 1:**
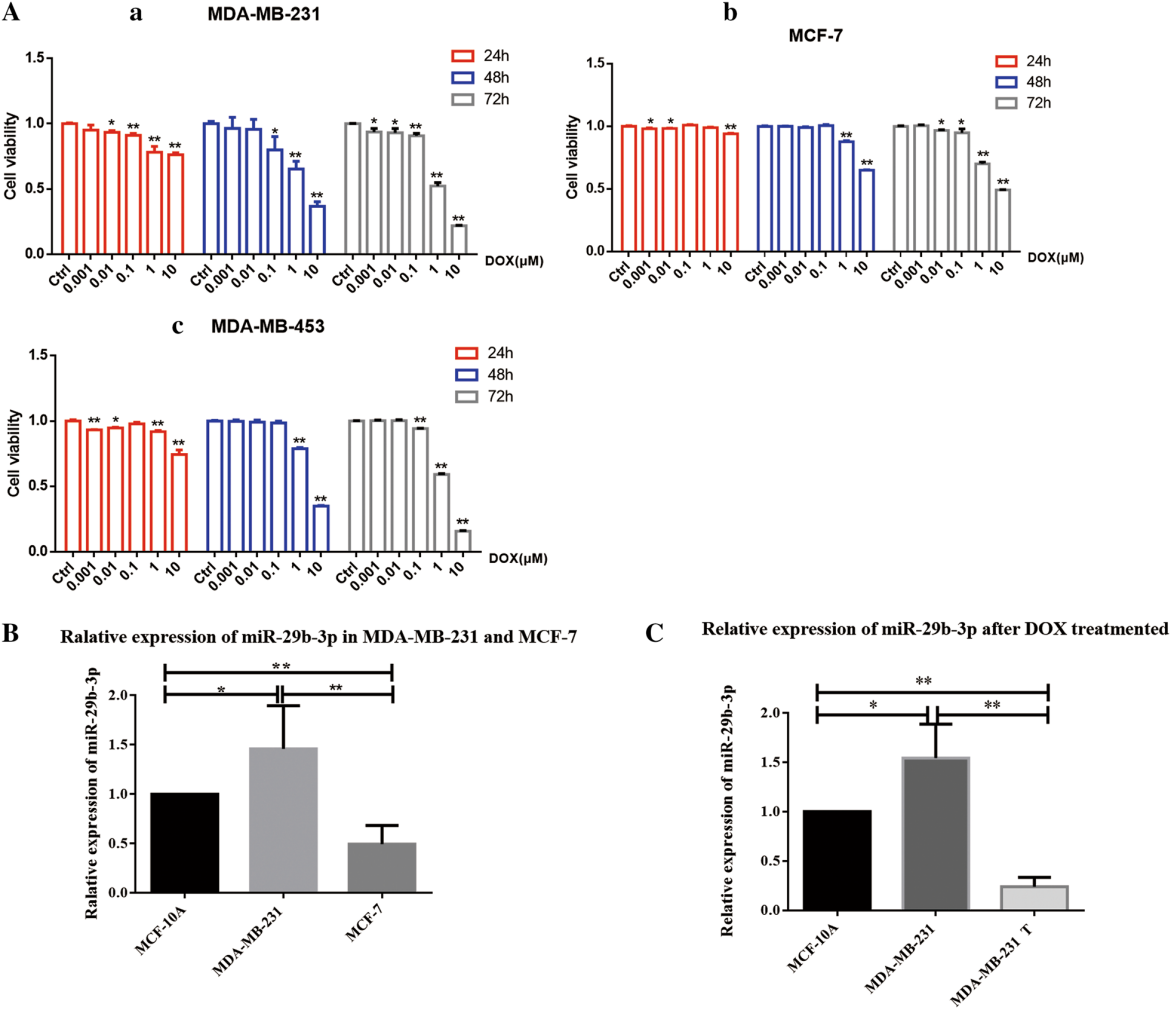
The expression of miR-29b-3p in breast cancer cells. **A** Doxorubicin exhibits cytotoxic effect in breast cancer cells. (a) MDA-MB-231, (b) MCF-7 and (c) MDA-MB-453. The percentage inhibition of cell viability represented graphically. **B** Analysis of miR-29b-3p expression level in MDA-MB-231 and MCF-7 cells compared with MCF-10A cells. **C** Analysis of miR-29b-3p expression level in MDA-MB-231 after Doxorubicin treated. U6 was used as an internal control. *T* treated. Data are presented as the mean ± SD of three independent experiments. *p < 0.05, **p < 0.01

In order to identify the relative expression of miR-29b-3p between different breast cancer cell lines, we performed qRT-PCR in MDA-MB-231, MCF-7 and human epithelial cell line MCF-10A. The results show that miR-29b-3p was over-expressed significantly in TNBC cell line compared to ER negative cell line MCF-7 and MCF-10A cell line (Fig. 1B). We used Doxorubicin for different breast cancer cell lines and found that the cell viability was inhibited by it, which was concentration and time dependent (Fig. 1A). And after DOX treatment, the miR-29b-3p was down-regulated in TNBC compare with before (Fig. 1C), which indicated that DOX regulated miR-29b-3p expression and miR-29b-3p may plays an important role in cell survival.

### MiR-29b-3p promotes TNBC cell proliferation in vitro

**Fig. 2:**
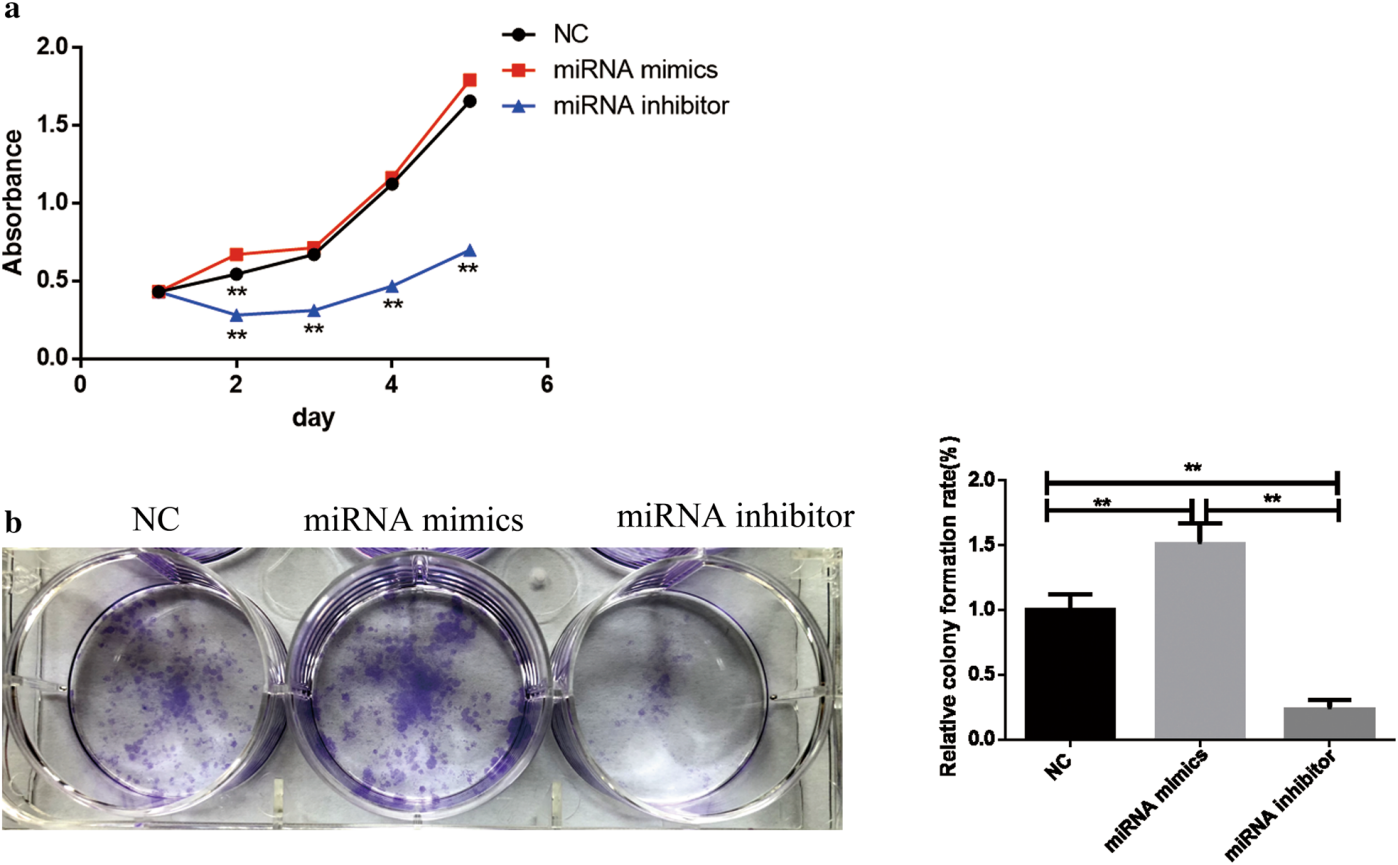
Cell proliferation and colony forming ability of MDA-MB-231 cells. **a** CCK-8 assay was applied to determine the activity of cells after transfection. **b** miR-29b-3p inhibitor inhibited cell colony formation in MDA-MB-231 cells. Data are presented as the mean ± SD of three independent experiments. *p < 0.05, **p < 0.01

To further investigate the biological function of miR-29b-3p in TNBC. We performed CCK8 and colony formation assay to detect the cell viability. After transfection CCK8 assay was performed to show the influence of miR-29b-3p to TNBC. The results indicated that miR-29b-3p inhibitor inhibit the growth rate significantly compare with NC and miR-29b-3p mimics group. Besides, colony formation assay showed that miR-29b-3p inhibitor could effectively reduce the cell viability of MDA-MB-231, resulting in less cell colony formation than NC and miR-29b-3p mimics group (Fig. 2).

### miR-29b-3p promotes MDA-MB-231 cell migration and invasion

**Fig. 3:**
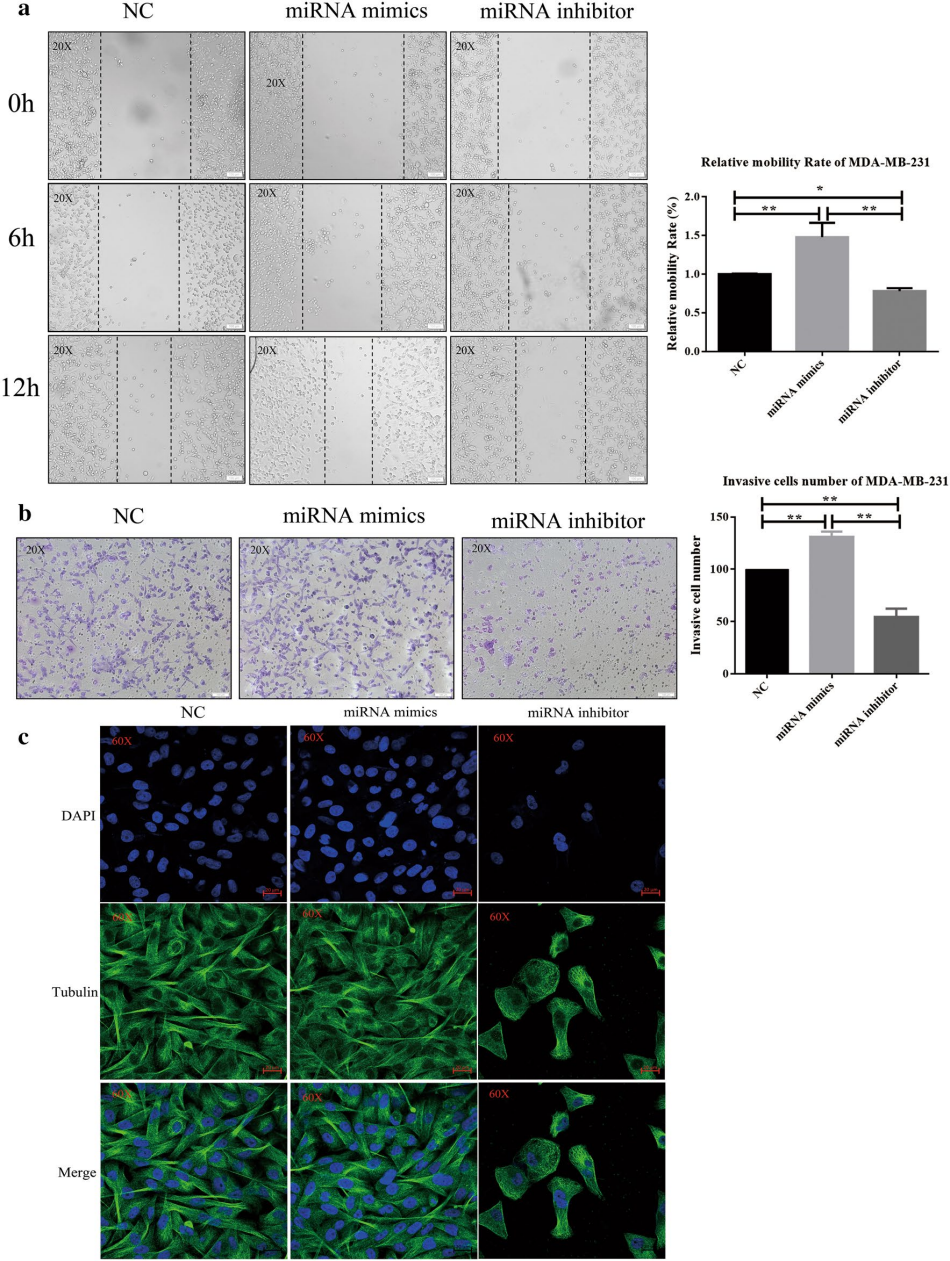
Cell migration and invasion of MDA-MB-231 cells. **a** miR-29b-3p inhibitor inhibited cell migration in the MDA-MB-231 cell lines. Image magnification: ×20. **b** miR-29b-3p inhibitor inhibited cell invasion in the MDA-MB-231 cell lines. Image magnification: ×20. **c** Immunofluorescence for cytoskeleton. The nuclei were visualized by staining with DAPI (blue). α-Tubulin (green). Image magnification: ×60. Data are presented as the mean ± SD of three independent experiments. *p < 0.05, **p < 0.01

MDA-MB-231 is highly aggressive cell line which exhibited a high migration and invasive capability. To assess the effects of miR-29b-3p on cell migration and invasion, migration assay and invasion assay were performed. The results suggested that overexpression of miR-29b-3p significantly promotes cell migration whereas suppressing the miR-29b-3p expression inhibits cell migration process (Fig. 3a). Comparable results were obtained from the invasion assay. The number of invasive cells significantly decreases in miR-29b-3p inhibitor group compared with NC and miR-29b-3p mimics group (Fig. 3b). The quantified results showed that miR-29b-3p inhibitor inhibits cell migration and invasion in MDA-MB-231. To study the cell integrity in the presence of miR-29b-3p inhibitor treatment we stained cells with the anti-α-tubulin antibody. The results revealed that miR-29b-3p inhibitor treatment damages the cytoskeleton and destroy cell integrity and probably affecting cell mobility (Fig. 3c).

### Inhibition of miR-29b-3p induces apoptosis in TNBC breast cancer cell line

**Fig. 4:**
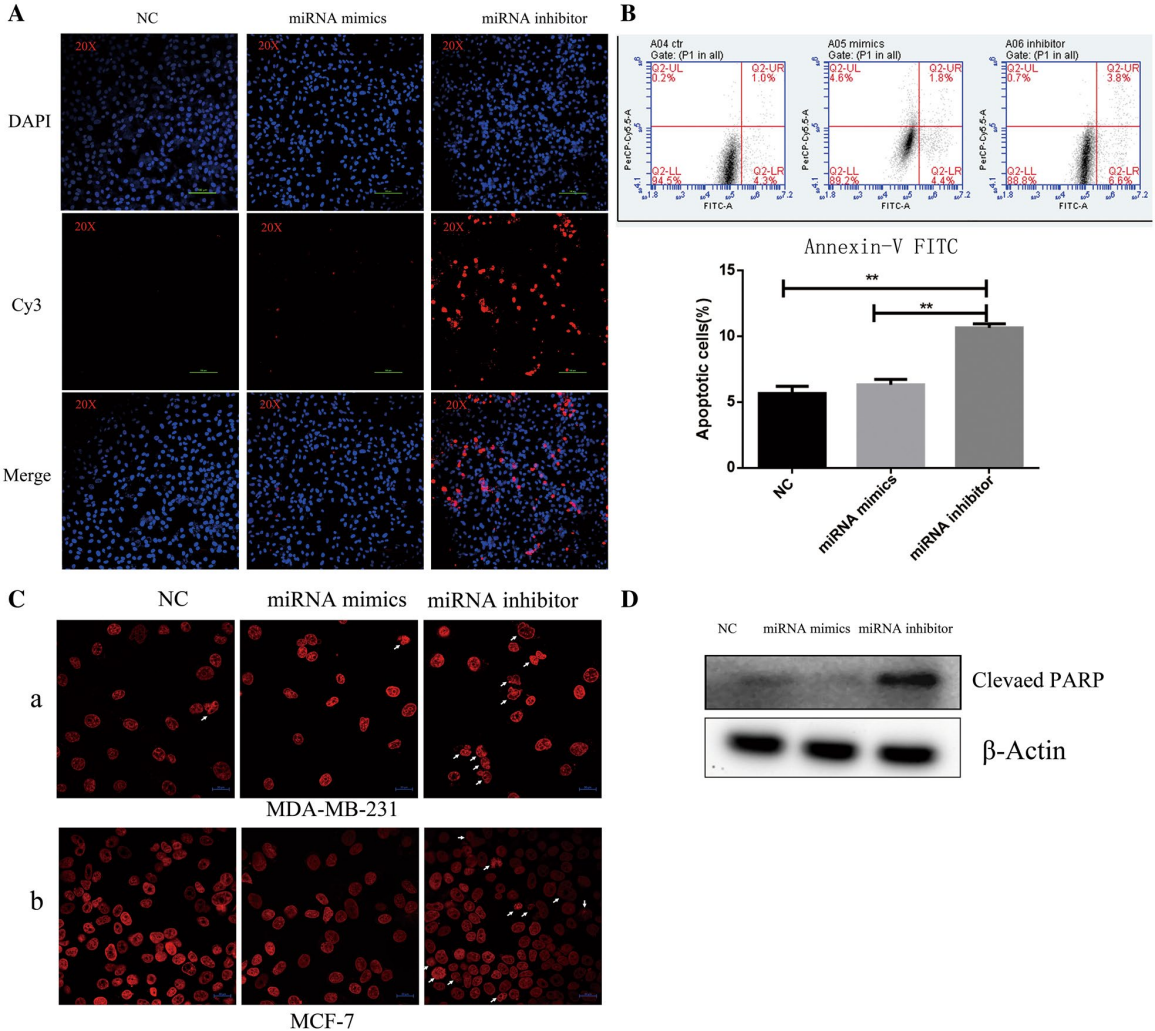
miR-29b-3p inhibitor induces apoptosis. **A** TUNEL assay. The nuclei were visualized by staining with DAPI (blue), Cy3-labeled the apoptosis cells (red). Image magnification: ×20. **B** Annexin V-FITC and propidium iodide assay. The sum of early and late apoptotic cells ratio (%) were quantitated by flow cytometer analysis of Annexin V/PI. **C** Hoechst staining. (a) MDA-MB-231; (b) MCF-7. White arrows indicate the cells nuclear fragmentation. The nuclei were visualized by staining with Hoechst (red). Image magnification: ×40. **D** Western blot for Cleaved PARP. Data are presented as the mean ± SD of three independent experiments. The data represents three experiments exhibiting similar results

To evaluate the apoptosis induced by miR-29b-3p inhibitor in MDA-MB-231, we performed TUNEL assay, Annexin V-FITC/PI staining, nuclear staining and Western Blot. MDA-MB-231 cells were transiently transfected with NC, miR-29b-3p mimics and miR-29b-3p inhibitor and stained with probe labelled with Cy3. As shown in Fig. 4A, fluorescence of Cy3 labeled probe was more in miR-29b-3p inhibitor group than NC and miR-29b-3p mimics group. In addition, Annexin V-FITC/PI staining was performed to determine direct effects of miR-29b-3p mimic and miR-29b-3p inhibitor on MDA-MB-231 breast cancer cells (Fig. 4B). The results showed that percentage of cell apoptosis in miR-29b-3p inhibitor group increased compare with NC and miR-29b-3p mimics group. To examine the nuclear morphology of apoptotic cells nuclear staining was conducted using Hoechst staining. The results showed that miR-29b-3p inhibitor increases the nuclear fragmentation and cell blebbing in MDA-MB-231 and MCF-7 cells (Fig. 4C). Further we isolated the total proteins and performed Western blot for poly ADP-ribose polymerase (PARP). Compared with the NC group, the miR-29b-3p inhibitor group exhibited an increased protein level of cleaved PARP while expression level in miR-29b-3p mimics group was less (Fig. 4D). Taken together, inhibition of miR-29b-3p will induce apoptosis in MDA-MB-231.

### miR-29b-3p targets to 3′-UTR of TRAF3 and regulates NF-κB signaling pathway in breast cancer

**Fig. 5:**
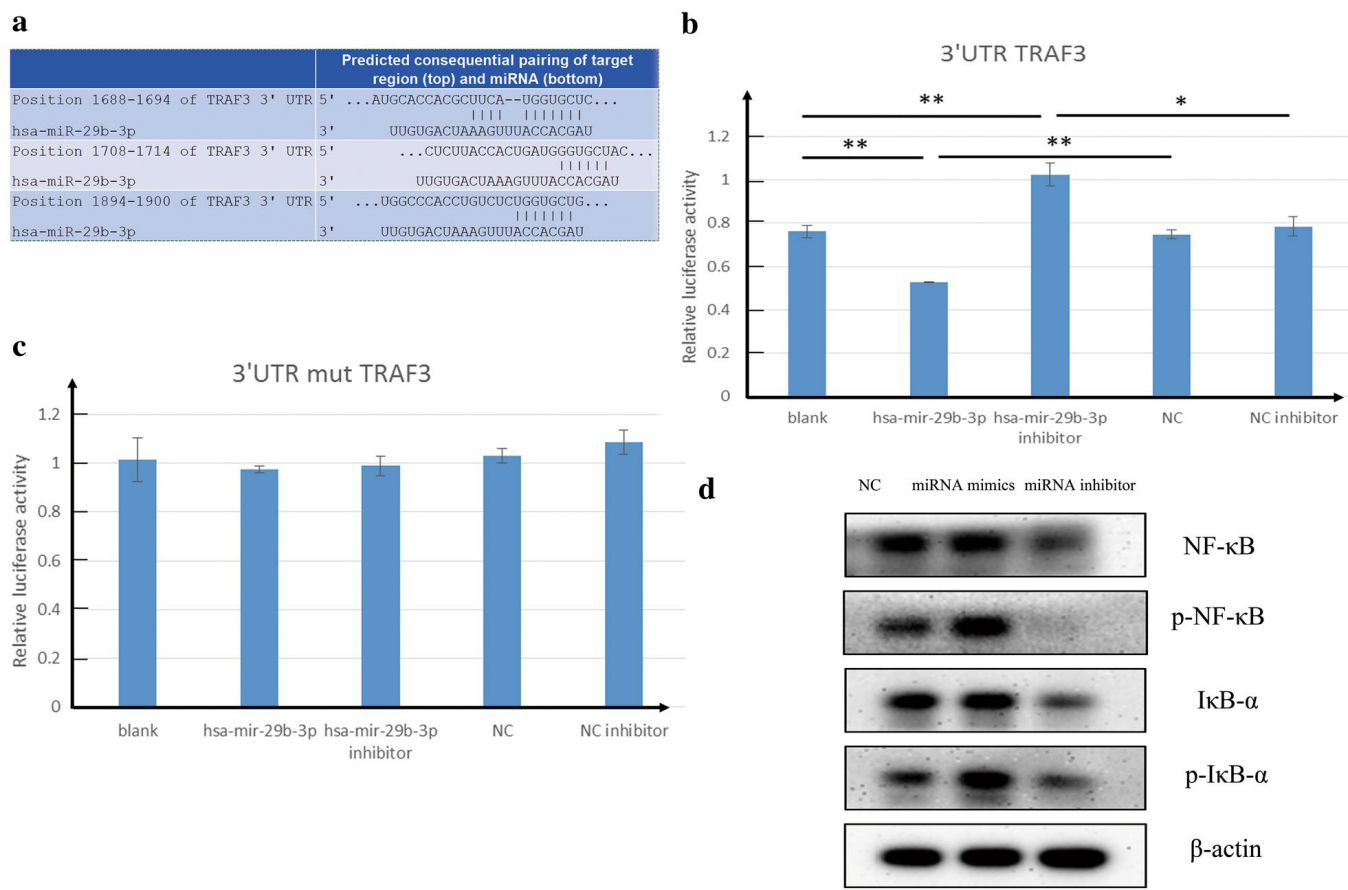
miR-29b-3p targets TRAF3 and Effects on NF-κB signaling pathway. **a** Alignment of the miR-29b-3p seed sequence with the TRAF3 3′ UTR. **b** TRAF3 3′-UTR wt relative luciferase activity. **c** TRAF3 3′-UTR mutant relative luciferase activity. **d** Western blot for NF-κB pathway. β-Actin was used as used as an internal control. Data are presented as the mean ± SD of three independent experiments. *p < 0.05, **p < 0.01

To identify the putative mechanisms of miR-29b-3p mediated regulation of MDA-MB-231, bioinformatics predictions were performed by using Targetscan, PicTar, miRDB. We found that miR-29b-3p targets to TRAF3 (Fig. 5a). Subsequently, Dual-luciferase reporter assays were performed to determine the relative luciferase activity in NC, miR-29b-3p mimics and miR-29b-3p inhibitor with TRAF3 3′-UTR wt or TRAF3 3′-UTR mt reporter plasmids. As shown in Fig. 5b, c, significant decrease in relative luciferase activity was noted when psi-CHECK2/TRAF3 3′-UTR wt was co-transfected with the miR-29b-3p mimics, while the relative luciferase activity in miR-29b-3p mimic group with psi-CHECK2/TRAF3 3′-UTR mt reporter plasmid had no significant difference with the NC group with psi-CHECK2/TRAF3 3′-UTR mt. Moreover, the relative luciferase activity in miR-29b-3p inhibitor group with TRAF3 3′-UTR wt reporter plasmid was markedly higher than the NC group with TRAF3 3′-UTR wt, while the relative luciferase activity in miR-29b-3p inhibitor group with TRAF3 3′-UTR wt reporter plasmid had no significant difference with the NC group with TRAF3 3′-UTR wt. Therefore, TRAF3 is one target gene of miR-29b-3p. To further understand the mechanism involved in miR-29b-3p regulating NF-κB pathway we performed Western blot experiment. As shown in Fig. 5d, NF-κB, p-NF-κB, IκB-α, p-IκB-α were decreased after miR-29b-3p inhibitor treatment whereas the miR-29b-3p mimics group was increased significantly compared with NC group. It indicated that miR-29b-3p induced activation of NF-κB signaling pathway and apoptosis resistance.

## Discussion

Breast cancer is a common malignant cancer among women worldwide. Triple negative breast cancer is more aggressive and has a higher mortality rate due to lack of effective treatments. Cumulative studies have demonstrated that miRNAs are molecular regulators in different diseases [33–36]. Our results found that miR-29b-3p was overexpressed in MDA-MB-231 while low-expressed in MCF-7 in compassion to MCF-10A. To explore the reasons behind this phenomenon, we used Doxorubicin (DOX), a well-known antitumor drug as positive control to induce apoptosis [37] in MDA-MB-231 cells and performed qRT-PCR. After DOX treatment, the miR-29b-3p expression was decreased significantly compare with non-treated MDA-MB-231 cells. The results indicated that miR-29b-3p may be involved in the progression and survival of breast cancer. In order to investigate the biological relevance of miR-29b-3p in breast cancer. miR-29b-3p inhibitor was transfected into MDA-MB-231 cells. Our results suggested that miR-29b-3p inhibitor inhibits cell proliferation, migration and invasion significantly in vitro. Cancer cell migration and invasion are responsible for the most number of cancer incidents [38, 39]. In this process, cancer cells acquired migratory and invasive capabilities and then migrator to distant site, this spreads the cancer cells throughout the body to seed secondary tumors at distant sites. It’s a complex process that involved a dramatic reorganization of cell cytoskeleton [40, 41]. Cell cytoskeleton refers to the protein fiber network system in eukaryotic cells which composed of microtubule (MT), microfilament (MF) and intermediate filament (IF). It has been reported that alterations in the cell cytoskeleton was associated with metastasis, invasion and apoptosis [42–45]. Herein, we performed immunofluorescence to check the formation of microtubule, our results showed that miR-29b-3p inhibitor has an influence on the formation of the microtubule which destroys cell integrity and probably affecting cell mobility, Consequently, miR-29b-3p is a tumor promoter in TNBC.

**Fig. 6:**
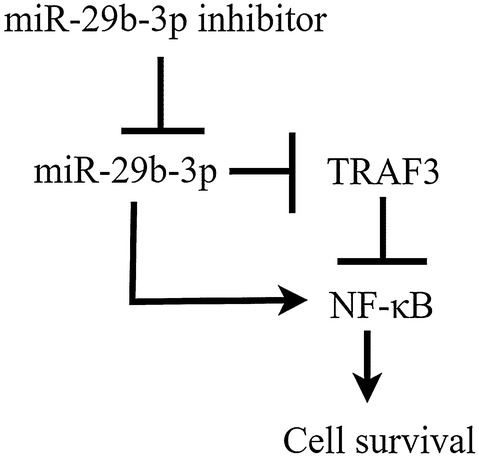
Schematic representation of miR-29b-3p inhibitor regulated miR-29b-3p mediated NF-κB pathway

Next, we predicated and validated the possible target mRNAs of miR-29b-3p. Previous studies have shown that miR-29b-3p targets many genes and exerts different biological functions, such as MCMBP, PI15, COL3A1 (TargetScan v7.2; http://www.targetscan.org). Therefore, based on the TargetScan predictions, it showed that TRAF3 is targeted by miR-29b-3p. Dual-luciferase reporter assay showed that significant decrease in relative luciferase activity was noted when psi-CHECK2/TRAF3-3′-UTR was co-transfected with the miR-29b-3p mimics compare with NC group, it indicated that miR-29b-3p mimics bind to the 3′UTR of the TRAF3, thereby inhibiting the luciferase activity. However, the relative luciferase activity was increased when psi-CHECK2/TRAF3 3′-UTR was co-transfected with the miR-29b-3p inhibitor compare with NC group. It indicated that miR-29b-3p inhibitor inhibits the endogenous miR-29b-3p binding to the TRAF3 3′-UTR, resulting in stronger relative luciferase activity than the NC group. But in miR-29b-3p mimics group, the relative luciferase activity was decreased compare with NC group. Moreover, no significant difference among groups when psi-CHECK2/TRAF3 3′-UTR mutant co-transfected with NC, miR-29b-3p mimics and miR-29b-3p inhibitor. The results showed that miR-29b-3p binds to TRAF3 and miR-29b-3p inhibitor inhibited miR-29b-3p. Therefore, TRAF3 is one target gene of miR-29b-3p. TRAF3, one of the six members of this family of proteins in humans and mice, has been reported to interact with other members of the TNFR family [46, 47] and mediate the signal transduction, such as CD40 and LMP-1. These two members are important for the activation of the immune response [48]. Different from many other TRAF family proteins that enhance NF-κB activation, TRAF3 has been reported to inhibit the activation of NF-κB induced by TNFR family [49] and was identified as a negative regulator of NF-κB inducing kinase (NIK), promoting its degradation [50, 51]. Activation of NF-κB signaling pathway is mediated by TNFR and it involved many biologic processes, such as cell proliferation, apoptosis and immune responses [52, 53]. During apoptosis, NF-κB signaling pathway is inhibited and expression of Bcl-2 is downregulated mediating the mitochondrial apoptotic pathway which release cytochrome C followed by caspase 9 and caspase 3 activation ultimately leading to PARP cleavage [54]. In accordance with the above studies, our results showed that miR-29b-3p inhibitor induces apoptosis and DNA fragmentation in MDA-MB-231, it suppresses NF-κB signaling pathway and induces PARP cleavage. Besides, over-expression of miR-29b-3p in TNBC resulting in inhibition of TRAF3 and activation of NF-KB signaling pathway. However, miR-29b-3p inhibitor can inhibit miR-29b-3p binding to TRAF3 (Fig. 6).

Taken together, we identified miR-29b-3p as an important regulator in TNBC which enhances cell proliferation, migration and invasion. Moreover, inhibition of miR-29b-3p induces cell apoptosis by suppressing NF-κB signaling pathway in MDA-MB-231. Altogether our results showed that miR-29b-3p has a key role in MDA-MB-231.

## Conclusion

Our findings indicated that miR-29b-3p was highly expressed in MDA-MB-231 cells. Inhibition of miR-29b-3p could suppress the cancer cells proliferation and induced the apoptosis. In addition, it influenced the formation of microtubule and suppressed the process of cell migration and invasion. Moreover, miR-29b-3p targets to the 3′-UTR of TRAF3 and activates the NF-κB signaling pathway. Therefore, miR-29b-3p could be an underlying biomarker and it can be developed as novel therapeutic target against the TNBC.

## Data Availability

Please contact the corresponding author for data on reasonable request.

## Abbreviations

miRNAs: microRNAs
NF-κB: nuclear factor kappa B
3′-UTR: 3′-untranslated region
TNBC: triple negative breast cancer
qPCR: quantitative PCR
DMEM: Dulbecco’s modified Eagle medium
FBS: fetal bovine serum
PARP: poly ADP-ribose polymerase
HRP: horse radish peroxidase
TRAF3: TNF receptor-associated factor 3
SD: standard deviation
